# Healthy Organs Uptake on Baseline ^18^F-FDG PET/CT as an Alternative to Total Metabolic Tumor Volume to Predict Event-Free Survival in Classical Hodgkin's Lymphoma

**DOI:** 10.3389/fmed.2022.913866

**Published:** 2022-06-22

**Authors:** David Morland, Elizabeth Katherine Anna Triumbari, Elena Maiolo, Annarosa Cuccaro, Giorgio Treglia, Stefan Hohaus, Salvatore Annunziata

**Affiliations:** ^1^Unità di Medicina Nucleare, TracerGLab, Dipartimento di Diagnostica per Immagini, Radioterapia Oncologica ed Ematologia, Fondazione Policlinico Universitario A. Gemelli, IRCCS, Roma, Italy; ^2^Service de Médecine Nucléaire, Institut Godinot, Reims, France; ^3^Laboratoire de Biophysique, UFR de Médecine, Université de Reims Champagne-Ardenne, Reims, France; ^4^CReSTIC (Centre de Recherche en Sciences et Technologies de l'Information et de la Communication), EA 3804, Université de Reims Champagne-Ardenne, Reims, France; ^5^Unità di Ematologia, Dipartimento di Diagnostica per Immagini, Radioterapia Oncologica ed Ematologia, Fondazione Policlinico Universitario A. Gemelli, IRCCS, Roma, Italy; ^6^Unità di Ematologia, ASL Toscana N/O Spedali Riuniti Livorno, Livorno, Italy; ^7^Clinic of Nuclear Medicine, Imaging Institute of Southern Switzerland, Ente Ospedaliero Cantonale, Bellinzona, Switzerland; ^8^Faculty of Biomedical Sciences, Università della Svizzera italiana, Lugano, Switzerland; ^9^Faculty of Biology and Medicine, University of Lausanne, Lausanne, Switzerland; ^10^Section of Hematology, Department of Radiological Sciences, Radiotherapy and Hematology, Università Cattolica del Sacro Cuore, Roma, Italy

**Keywords:** cerebellum, liver, metabolic tumor volume, Hodgkin's Lymphoma, prognosis, prediction

## Abstract

**Purpose:**

Healthy organs uptake, including cerebellar and liver SUVs have been reported to be inversely correlated to total metabolic tumor volume (TMTV), a controversial predictor of event-free survival (EFS) in classical Hodgkin's Lymphoma (cHL). The objective of this study was to estimate TMTV by using healthy organs SUV measurements and assess the performance of this new index (UF, Uptake Formula) to predict EFS in cHL.

**Methods:**

Patients with cHL were retrospectively included. SUV values and TMTV derived from baseline ^18^F-FDG PET/CT were harmonized using ComBat algorithm across PET/CT systems. UF was estimated using ANOVA analysis. Optimal thresholds of TMTV and UF were calculated and tested using Cox models.

**Results:**

163 patients were included. Optimal UF model of TMTV included age, lymphoma maximum SUVmax, hepatic SUVmean and cerebellar SUVmax (R^2^ 14.0% - *p* < 0.001). UF > 236.8 was a significant predictor of EFS (HR: 2.458 [1.201–5.030], *p* = 0.01) and was not significantly different from TMTV > 271.0 (HR: 2.761 [1.183–5.140], *p* = 0.001). UF > 236.8 remained significant in a bivariate model including IPS score (*p* = 0.02) and determined two populations with different EFS (63.7 vs. 84.9%, *p* = 0.01).

**Conclusion:**

The Uptake Formula, a new index including healthy organ SUV values, shows similar performance to TMTV in predicting EFS in Hodgkin's Lymphoma. Validation cohorts will be needed to confirm this new prognostic parameter.

## Introduction

Hodgkin's Lymphoma (HL) affects young adults and represents about 2.3 cases per 100,000 people per year, with an associated mortality of 0.4 cases per 100,000 per year (1). Despite treatment, about 20% of HL patients still relapse (1). ^18^F-Fluorodeoxyglucose (^18^F-FDG) Positron Emission Tomography (PET) coupled with Computed Tomography (CT) plays a central role in HL patients management, whether in staging or response assessment settings (1).

PET-derived parameters, volumetric ones above all, have been proposed to refine prognosis prediction of HL (2). The role of Total Metabolic Tumor Volume (TMTV) is debated in Hodgkin's Lymphoma. It has been reported as a negative prognostic factor in early-stage HL treated with ABVD (Adriamycin, Bleomycin, Vinblastine, Dacarbazine) regimen (3–5) and HIV-associated HL (6). However, some publications reported no association between TMTV and Progression-Free Survival (PFS) in advanced-stage HL when treated with escalated BEACOPP (7). Furthermore, TMTV threshold varies from one study to another (3–5, 7, 8).

The drawbacks of TMTV calculation (results depending on the segmentation method (9), time required for delineation (10), difficulty in evaluating bone involvement) led to ponder other prognostic markers, such as healthy organ ^18^F-FDG uptake. In 2010, Hanaoka et al. (11) reported an inverse correlation between cerebellar uptake and total lesion glycolysis (TLG) in a population with aggressive lymphoma. The mechanism underlying this phenomenon is poorly understood but could correspond to a metabolic theft of ^18^F-FDG by the tumor mass. Because TLG is correlated with TMTV, Godard et al. (12) speculated that cerebellar metabolism might have a prognostic value and suggested to normalize cerebellar ^18^F-FDG uptake to hepatic ^18^F-FDG uptake to account for differences between PET/CT systems. This index has been shown to be a prognostic parameter for PFS prediction in diffuse large-B-cell lymphoma (10) and follicular lymphoma (12). Normalization to liver was not optimal: liver ^18^F-FDG uptake was also negatively correlated with TMTV as was cerebellar ^18^F-FDG uptake (*r* = −0.34 and *r* = −0.42, respectively) (10). These two healthy organs could thus prove useful in predicting prognosis.

The objective of this study was to model TMTV by integrating healthy organ uptake data in classical HL (cHL). The resulting estimate was then tested for EFS prediction.

## Materials and Methods

### Study Population

This study was approved by the Ethical Committee of Fondazione Policlinico Universitario A. Gemelli IRCCS (study code: 3834). All included subjects signed an informed consent form. All procedures performed were in accordance with the ethical standards defined by the 1964 Helsinki Declaration and its later amendments.

All patients with HL referred to our Institution for their baseline ^18^F-FDG PET/CT between September 2010 and January 2020 were retrospectively screened. Inclusion criteria were as follows: histologically proven cHL; baseline PET/CT performed within 4 weeks prior to treatment. Exclusion criteria were: Recent history of other cancer <1 year; Nodular lymphocyte-predominant Hodgkin lymphoma histology [slow growing LH subtype with completely different prognosis (13)]; any factor interfering with measurement of cerebellar uptake, liver uptake or TMTV: cerebellum not fully included in field of view, movement artifacts, extensive surgically resected disease before staging PET/CT, diffuse lymphomatous involvement of liver or brain lymphoma; nonobservance of a fasting period of at least 6h before ^18^F-FDG administration; glycemia > 2.0 g/l; no follow-up available after staging PET/CT; nonstandard treatment regimen.

The following clinicobiological data were collected: date of birth, date of diagnosis, date of last observation, HL subtype, Eastern Cooperative Oncology Group (ECOG) performance status, International Prognostic Score (IPS) items (age; sex; Ann Arbor stage; serum albumin levels, white blood cell count, lymphocyte count, hemoglobinemia at baseline), first-line treatment, event-free survival (EFS: time interval between date of diagnosis and the event of progression, recurrence, change of therapy or death) and overall survival (OS: time interval between date of diagnosis and death). Imaging data collected included: date of staging PET/CT, PET/CT system, administered ^18^F-FDG activity, glycemia levels.

### ^18^F-FDG PET/CT Acquisition and Measurements

After verification of patients' blood glucose levels, baseline ^18^F-FDG PET/CT was performed at 60 ± 10 min after intravenous injection of mean 236.34 MBq (range 137–366) of ^18^F-FDG. Due to the long-time span of the inclusion period, images were acquired using 3 different PET/CT integrated systems denoted as PET1, PET2 and PET3 in chronological order.

PET1 corresponded to a Gemini Dual GS PET/CT scanner (Philips Healthcare): images were acquired in three-dimensional mode with an acquisition time of 3 min per bed position and reconstructed on a 128 × 128 matrix using Row-Action Maximum Likelihood Algorithm (RAMLA, 2 iterations, blob size of 2.5 pixels, voxel size: 4 × 6 × 20 mm^3^) without Point Spread Function (PSF) or Time of Flight (TOF).

PET2 corresponded to a Gemini GXL PET/CT scanner (Philips Healthcare): images were acquired with a 3 min per bed position acquisition time and reconstructed on a 128 × 128 matrix using 3D-Line Of Response RAMLA (3D-LOR-RAMLA, 3 iterations, 33 subsets, voxel size: 4 × 4 × 4 mm^3^) without PSF or TOF.

PET3 corresponded to a Biograph mCT PET/CT scanner (Siemens Healthineers): images were acquired in 2.5 min per bed position and reconstructed on a 400 × 400 matrix using 3D Ordered Subset Expectation Maximization algorithm (3D-OSEM, 2 iterations, 21 subsets, voxel size: 3.2 × 3.2 × 5 mm^3^) with PSF and TOF. A gaussian filter was also applied (3D isotropic Gaussian kernel of 2 mm full width at half-maximum).

CT acquisition protocol was the same for the three machines: 120 kV, 50 mAs, reconstruction on a 512 × 512 matrix with a voxel size of 0.97 × 0.97 × 3 mm^3^. PET/CT images were acquired at least from skull base to proximal thighs.

PET/CT were displayed on dedicated interpretation consoles (Syngo.via for SUV measurements and version 7.0.5 of MIM Encore Software for volumetric parameters). The following data were collected: (1) cerebellar SUV_max_, (2) hepatic SUV_mean_, (3) TMTV, (4) lymphoma maximal SUV_max_ (lesion SUV_max_, henceforth denoted as L).

After a first visual check using a rainbow 10 point-color scale, enclosing region of interest (ROI) were drawn on areas with highest visual uptake, excluding any voxel of the neighboring brain hemispheres. The highest SUV_max_ of all these ROI corresponded to cerebellar SUV_max_. A default spherical ROI (2-cm diameter) was positioned on the right liver to measure its SUV_mean_. The lymphoma maximal SUV_max_ was determined manually by an experienced nuclear medicine physician and was defined as the SUV_max_ of the hottest nodal lesion. TMTV was measured using a PET segmentation tool (LesionID, version 7.0.5 of MIM Encore Software Inc., Cleveland, OH). As previously described (14), the software proceeds in 4 steps: first, a PET Response Criteria in Solid Tumors (PERCIST)-based background threshold (liver) thresholding was applied; second, a VOI encompassing all detected lesions (above the threshold) was automatically drawn. The detected lesions could thus include bone and spleen, depending on their uptake; third, a second thresholding at 41% of the SUVmax of the detected lesions was applied to determine lesions' boundaries; finally, physicians were required to reject false positive lesions before computation of TMTV.

### ComBat Harmonization

**Figure 1:**
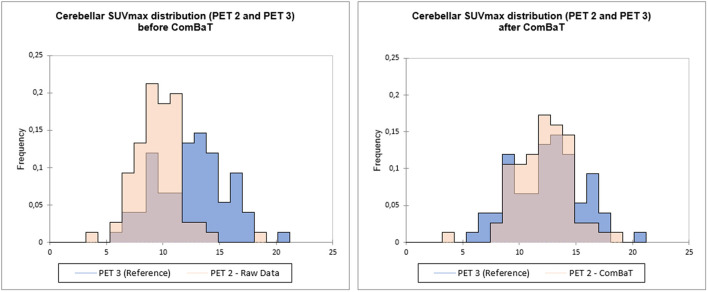
Example of ComBaT harmonization. Cerebellar SUVmax distribution of PET2 before **(left)** and after ComBaT harmonization **(right)**, PET3 (blue) being the reference.

The “batch effect” introduced by the use of 3 different PET/CT machines was compensated using a validated statistical harmonization method (15) implemented on RStudio (16). ComBat was applied on log transformed data, followed by exponentiation to improve the algorithm effectiveness (15), and ensure positive values. TMTV, hepatic SUV_mean_ (H), cerebellar SUV_max_ (C) and lesion SUV_max_, (L) were harmonized. Reference batch was set to PET3. An example is presented in Figure 1.

### Statistical Analyses

#### Statistical Software

If not stated otherwise, the following statistical analyses were performed on Xlstat (2020, Addinsoft, New York, USA). *p*-value threshold for significancy was set at 0.05.

#### TMTV Modelization

An ANOVA analysis was used to model the TMTV from the following 5 clinicobiological data: age, blood glucose, H, C, L. The selection of the optimal model was based on the R^2^ value with a number of allowed parameters ranging from 2 to 5. The resulting formula is hereafter referred to as the Uptake Formula (UF). Significance was assessed by F-statistic.

#### Analysis

Optimal cut-off for TMTV, UF and IPS were calculated using CutoffFinder (17) with respect to EFS using the survival analysis method. This method fits Cox proportional hazard models to the dichotomized variable and the survival variable: optimal cutoff is defined as the point with the most significant (log-rank test) split. Missing IPS values were replaced by mean-values. Derived Hazard Ratios were compared based on their 95% Confidence Intervals. Bivariate analysis was performed using TMTV+IPS and UF+IPS. TMTV and UF were not combined for collinearity issues. Survival curves were drawn for UF and TMTV.

## Results

**Figure 2:**
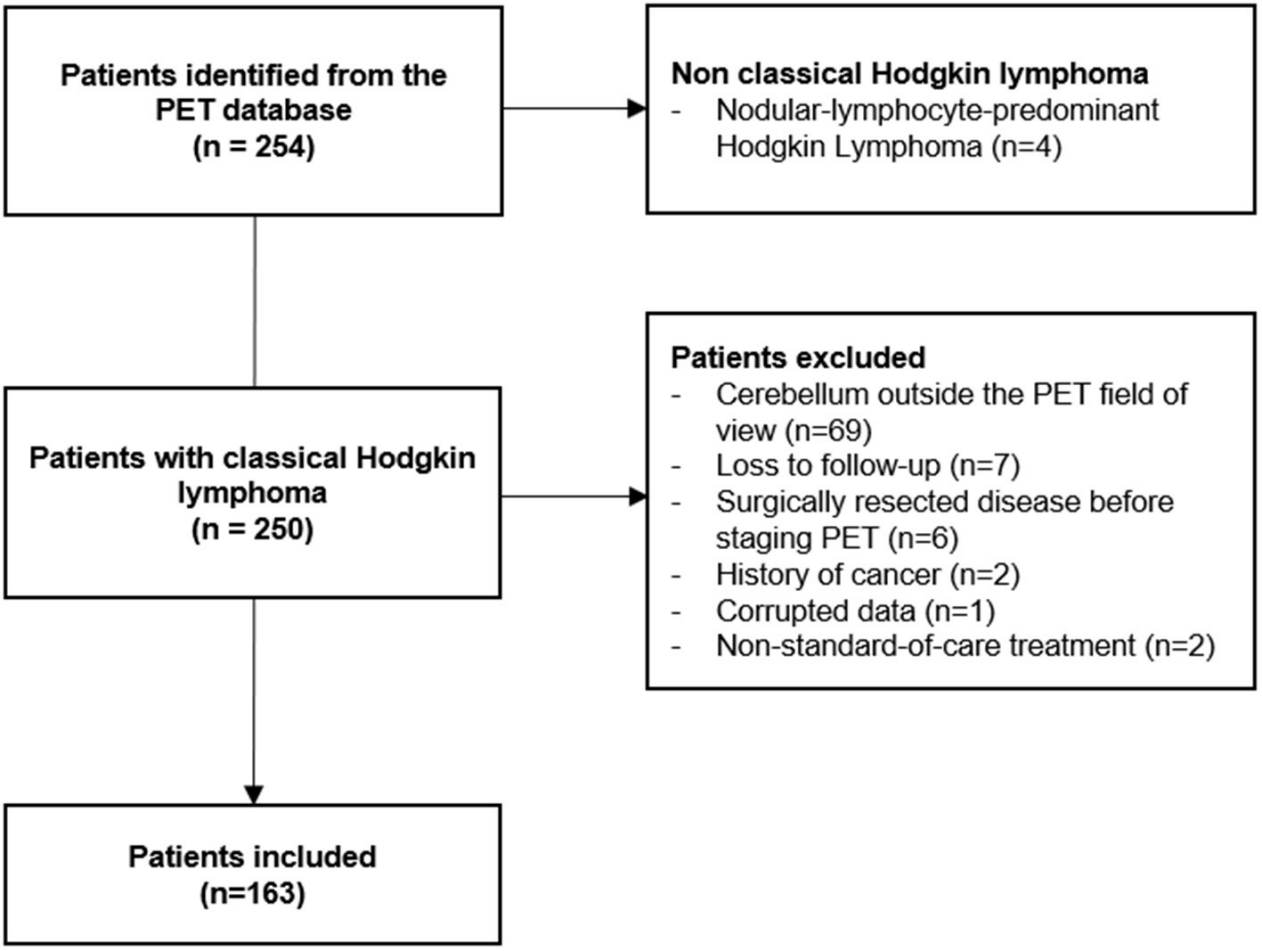
Flowchart of patients' selection.

Two-hundred and fifty-four patients were retrieved from the database (Figure 2). Among them, 4 had a nodular-lymphocyte-predominant HL (1.6%) and were excluded. Among the remaining 250 patients, 77 were excluded due to the impossibility of measuring the needed parameters (cerebellum outside the field of view, surgically resected disease, corrupted data). Seven patients were lost at follow-up just after the baseline PET. Two patients had a history of recent cancer. Two patients were treated with non-standard-of-care chemotherapy. Finally, 163 patients were included in the analysis. Their main characteristics are presented in Table 1. The median follow-up was 51 months (range 3–127 months). Overall, 9 patients died during follow-up (5.5%) and 40 EFS events were recorded (24.5%).

**Table 1:** Patients' characteristics.

	**Included patients (n=163)**
**Hodgkin's Lymphoma subtype**	
Nodular sclerosis	125 (76.7%)
Mixed cellularity	9 (5.5%)
Lymphocyte-rich	2 (1.2 %)
Lymphocyte-depleted	7 (4.3%)
Not specified	23 (14.1%)
**ECOG performance status**	
0	65 (39.9%)
1	41 (25.2%)
2	7 (4.3%)
3	2 (1.2%)
Not available	48 (29.4%)
**International Prognosis Score items**	
Age ≥ 45 years	51 (31.3%)
Male sex	77 (47.2%)
Ann Arbor stage IV	56 (34.4%)
Serum albumin <4 g/dl	78 (47.9%)
White Cell count ≥ 15,000/mm^3^	23 (14.1%)
Lymphocyte count <600/mm^3^	9 (5.5%)
Hemoglobin <10.5 g/dl	30 (18.4%)
**First-line chemotherapy treatment**	
ABVD	127 (77.9%)
ABVD + BEACOPP	18 (11.0%)
MBVD	10 (6.1%)
BEACOPP	5 (3.1%)
Not available	3 (1.8%)
Radiotherapy	134 (82.2%)
Number of EFS events	40 (24.5%)
Number of OS events	9 (5.5%)
**PET/CT systems**	
PET1	9 (5.5%)
PET2	70 (42.9%)
PET3	84 (51.5%)

*ABVD, Adriamycin, Bleomycin, Vinblastine, Dacarbazine; MBVD, nonpegylated liposomal doxorubicin (Myocet), Bleomycin, Vinblastine, Dacarbazine; BEACOPP, Bleomycin, Etoposide, Adriamycin, Cyclophosphamide, vincristine (Oncovin), Procarbazine, Prednisolone*.

### TMTV Modelization

ANOVA analysis selected 1 constant and 4 parameters to model TMTV (*R*^2^: 14.0%—*p* < 0.001): age, lesion SUV_max_ (L), cerebellar SUV_max_ (C), and hepatic SUV_mean_ (H). Coefficient values are presented in Table 2. Lesion SUV_max_ was a positive coefficient while healthy organs SUV values corresponded to negative coefficients. Glycemia was excluded.

**Table 2:** ANOVA analysis and derived model for TMTV prediction.

	**Coefficient (95%CI)**	***p*-value**
Constant	382.150 [181.543, 582.757]	<0.001^**^
Age (A)	−2.449 [−4.675, −0.223]	0.031^*^
Lesion SUVmax (L)	9.145 [4.263–14.026]	<0.001^**^
Cerebellar SUVmax (C)	−13.674 [−26.652, −0.695]	0.039^*^
Hepatic SUVmean (H)	−20.008 [−42.541, 2.526]	0.081
Glycemia (G)	Rejected	Rejected

* *p < 0.05*;

** *p < 0.001*.

The resultant UF was: *TMTV* = *382.150 – 2.449 Age* + *9.145 L – 13.674 C – 20.008 H*.

### Event-Free Survival Analysis

Optimal threshold for UF, TMTV and IPS were 236.8, 271.0, and 2.0, respectively (Table 3). The three parameters were significant predictors of EFS with HR between 2.050 and 2.761. When pooled with IPS, both UF and TMTV remained significant predictors of EFS (*p* = 0.022 and *p* = 0.004, respectively).

**Table 3:** Univariate and bivariate analyses for Event-Free Survival (EFS) based on Cox model.

	**Hazard Ratios (95% CI)**	***p*-value**
**UF** **>** **236.8**	2.458 [1.201–5.030]	0.014^*^
**TMTV** **>** **271.0**	2.761 [1.183–5.140]	0.001^*^
**IPS** **>=** **2**	2.050 [1.023, 4.106]	0.043^*^
**UF** **+** **IPS Model**
- UF	2.320 [1.131–4.760]	0.022^*^
- IPS	1.903 [0.947–3.822]	0.071
**TMTV** **+** **IPS Model**
- TMTV	2.507 [1.333–4.715]	0.004^*^
- IPS	1.732 [0.854–3.513]	0.128

* *p < 0.05*.

**Figure 3:**
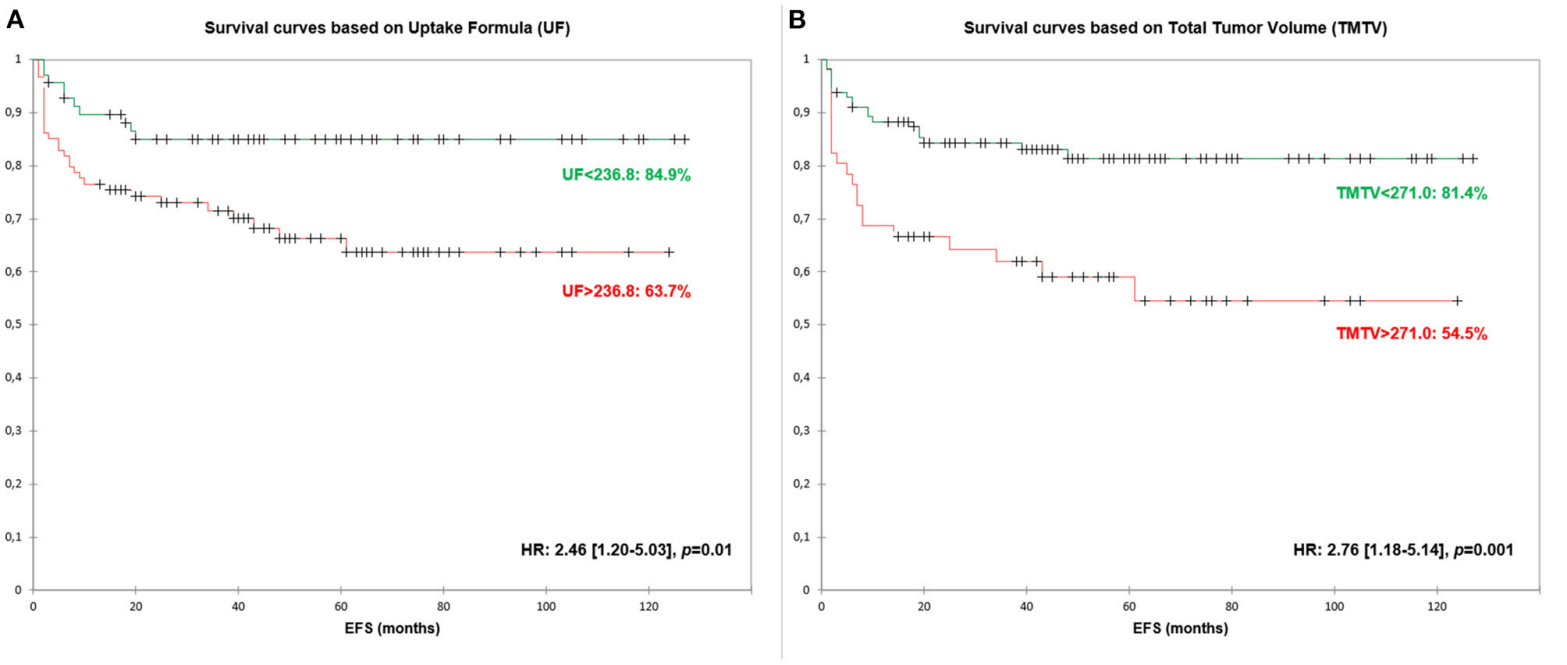
Event-Free Survival (EFS) curves based on Uptake Formula [UF—**(A)**] and Total Metabolic Tumor Volume [TMTV—**(B)**].

EFS survival curves based on UF are presented in Figure 3.

## Discussion

### Healthy Organs ^18^F-FDG Uptake Values and Derived Formula

No significant differences in hazard ratio were found between UF and TMTV. UF remained significant at bivariate analysis when adding IPS score with an HR of 2.3 (derived EFS of 84.9 vs. 63.7%).

The metabolic theft hypothesis for which FDG-avid tumor masses would deprive healthy organs of ^18^F-FDG was investigated in two papers on diffuse large-cell B-cell and follicular lymphomas (10, 12). Cerebellar and hepatic ^18^F-FDG uptake values were reported to be inversely correlated to TMTV. The optimal model we found to estimate TMTV is coherent with these findings: both liver and cerebellum coefficients are negative, meaning an inverse correlation with TMTV estimate. The addition of these two parameters contributed to the significance of the model, which, however, solves only part of the variability in TMTV (*R*^2^ of 14.0%), explaining the slight difference between TMTV and UF optimal thresholds.

Besides healthy organ uptake, age and tumoral ^18^F-FDG uptake were also selected in the model. Apart from the study by Angelopoulou et al. (18), who reported that SUV_max_ was predictive of PFS in a study of 162 patients with HL, other studies reported no significance (3). The lack of harmonization is probably one of the overriding factors for those results.

Both SUV_mean_ and SUV_max_ were used. SUV_mean_, which is less sensitive to noise, was preferred for the measurement of hepatic uptake to promote reproducibility: as the liver is a homogeneous organ, variations in the positioning of the ROI have little impact on SUVmean measurement as already noted in a previously published study (12). The cerebellum has on the other hand a heterogeneous uptake mainly concentrated in the gray matter. The measurement of SUVmean would have required a precise contouring of this structure that could have introduced contouring bias (19). SUVmax, which relies on only a single pixel was then chosen to ensure reproducibility, as already demonstrated in another study (12).

UF thus had the advantage of speed of calculation, requiring only 3 measurements of SUV values vs. several minutes for TMTV [6.2 min on average, ranging from 0.4 to 21.6 min in the study by Ilyas et al. (20)].

### Metabolic Tumor Volume as Prognostic Factor

Some studies have already investigated the prognostic value of TMTV in HL (3–7, 18, 21–24) with conflicting results, presumably related to the difference in patients' therapeutic management and the low number of events encountered in HL (25). Most studies reported a significant ability of TMTV to predict PFS (3, 5, 22), with an overall HR calculated by Frood et al. (25) of 2.13 (CI 95% 1.53–2.96). These results were however associated with high levels of heterogeneity. Segmentation methods and cut-offs varied greatly [TMTV cut-off from 89 ml (22) to 225 ml (21)] and no test-retest of these thresholds were performed. Moreover, the cut-off determination method is another aspect that needs to be addressed. As pointed out by Schöder and Moskowitz (26), most studies rely on Receiver Operating Curves to determine variables' cut-offs, neglecting censored data and leading to inappropriate results (27–29). To overcome this issue, a survival-based cut-off method was used in this study. Even if our TMTV threshold (271 cm^3^) was higher than the previously mentioned ones, we found a similar HR to previously reported ones.

### Harmonization

Pooling images from different scanners is not simple, as many quantitative biomarkers (SUV, TMTV) are sensitive to a scanner effect (15, 30). Although procedures were proposed to harmonize image quality (31), a dedicated reconstruction requiring raw data storage would be needed and would mostly be not feasible in a retrospective setting (15). To counteract this batch effect, the ComBat harmonization method, initially introduced in the field of genomics (32), has been proposed (15) and used (33). ComBat is a data-driven method that does not require phantom acquisitions to estimate the scanner effect but requires data from the different sites with sufficient sample size. It always theoretically improves the alignment of the mean and standard deviation of the distributions, given the criterion optimized by the method (15).

In our study, we chose to harmonize SUV and TMTV values using PET3 scanner as a reference for several reasons: it is the most recent machine among the three, corresponding to currently available technology in PET scanners; furthermore, the majority of patients was scanned on the PET3, so TMTV and SUV values were not modified for the majority of patients. Harmonization allowed us to study cerebellar and hepatic uptake independently, without having to use a ratio for normalization purposes. The use of a ratio disturbed the correlation between healthy organ and TMTV in the article by Morland et al. (10), but was still necessary to ensure good inter-machine agreement. The ComBat harmonization allowed us to overcome this problem.

### Limitations

Some limitations of this study can be pointed out. This exploratory retrospective study, lacks an external and/or prospective validation cohort which would be desirable to confirm our findings. Moreover, the cohort is heterogeneous due to different stages at diagnosis that may have interfered with the performance of the parameters tested. Nevertheless, large retrospective studies are commonly designed to evaluate prognostic parameters in lymphoma, needing long-term follow-up to register a significant number of adverse events.

## Conclusion

The Uptake Formula, a new index including healthy organ uptake values, shows similar performance to TMTV in predicting EFS in Hodgkin's Lymphoma. Validation cohorts will be needed to confirm this new prognostic parameter.

## Data Availability Statement

The raw data supporting the conclusions of this article will be made available by the authors, without undue reservation.

## Ethics Statement

The studies involving human participants were reviewed and approved by Ethical Committee of Fondazione Policlinico Universitario A. Gemelli IRCCS (Study Code: 3834). The patients/participants provided their written informed consent to participate in this study.

## Author Contributions

All authors listed have made a substantial, direct, and intellectual contribution to the work and approved it for publication.

## Funding

SA and ET were supported by the Italian Ministry of Health (GR-2019-12370372).

## Conflict of Interest

The authors declare that the research was conducted in the absence of any commercial or financial relationships that could be construed as a potential conflict of interest.

## Publisher's Note

All claims expressed in this article are solely those of the authors and do not necessarily represent those of their affiliated organizations, or those of the publisher, the editors and the reviewers. Any product that may be evaluated in this article, or claim that may be made by its manufacturer, is not guaranteed or endorsed by the publisher.
